# Eduard Gamper (1887–1938): cases and accidents

**DOI:** 10.1007/s00415-021-10795-0

**Published:** 2021-09-13

**Authors:** Evzen Ruzicka, Hartmann Hinterhuber, Hans Förstl

**Affiliations:** 1 Department of Neurology and Center of Clinical Neuroscience, I Medical Faculty, Charles University, Praha, Czech Republic; 2 Department für Psychiatrie, Psychotherapie und Psychosomatik, Medizinische Universität Innsbruck, Innsbruck, Austria; 3 Klinik für Psychiatrie und Psychotherapie, TU München, München, Germany

**Keywords:** Charles University, Gamper’s midbrain-being, Gamper’s reflex, Philipp Halsmann

## Abstract

Eduard Gamper (1887–1938) was Head of the Department of Neuropsychiatry at the Charles University’s German Faculty of Medicine in Prague. He had trained in Innsbruck, where he undertook groundbreaking work on the midbrain in an anencephalic child and in a series of patients who had died from Wernicke’s encephalopathy. Gamper identified the mamillary bodies as key in the performance of declarative memory. Considered an expert in memory disorders, he was chosen by the Medical Faculty in Innsbruck to provide expert opinion on the notorious case of Philipp Halsmann, who was suspected of murdering his father. Details of the case remained unresolved and Gamper’s opinion caused both professional and political controversy. When in Prague, Gamper made great efforts to improve patient care and clinical services, establishing a special ward for patients with neurological conditions. This task was not nearly completed, when he and his wife died after their car drove over a cliff into the Walchensee in Bavaria. Rumours surrounded his death: that Gamper had just examined Adolf Hitler; that he was a political victim; that the Gestapo were behind the accident. After an investigation of the available evidence, we can report that an unusual 22 cm of snow fell in the area of the Walchensee on April 20, 1938, the day of the Gampers’ deaths. We were unable to find any evidence for foul play in what appears to have been a tragic accident.

## Career

Eduard Gamper was born in Kappl in Tyrol on 23 June, 1887, the eldest of the eight children of Dr. Eduard Gamper senior, a General Pracitioner, and his wife Anna Maria, nee Rudigier [1, 2]. In 1892, the family moved to Reutte where Eduard attended elementary school for 5 years. He was an excellent student and progressed to the Vincentinum High School (Bischöfliches Gymnasium) in Brixen, where he obtained his baccalaureate “with distinction” in 1906. He began to study Medicine at the University of Innsbruck during the same year. Gamper worked as a teaching assistant in obstetrics with Professor Emil Ehrendorfer (1853–1945), for 2 years before he received his doctorate in 1911 [1–3]. After a brief period as a voluntary, he became assistant at the Department of Neuropsychiatry headed by Prof. Carl Mayer (1862–1936) at the University of Innsbruck, where he stayed until 1930. Gamper married Pierina Casper (born 1889) in 1913. She died after gall bladder surgery in 1918. During World War I, Gamper was considered indispensible and carried the brunt of the clinical work from the Department [1, 2, 4]. In 1920, he was made Lecturer in Neuropsychiatry. During the first half of 1925, he studied neuropathology with Walther Spielmeyer (1879–1935) and his group at the German Research Institute for Psychiatry in Munich with a Rockefeller fellowship. There he met his future wife, Helene Seyfarth (born 1895), whom he married in 1927 [1, 2, 5]. After his return to Innsbruck, Gamper became Head of the new laboratory of neuroanatomy and was made associate professor [1, 2, 5].

In December 1928, Gamper was elected to the Chair of Neuropsychiatry in the German Faculty of Medicine at the Charles University in Prague [6–8]. Oskar Vogt (1870–1959) had invited him to work at the Brain Research Institute in Berlin, but Gamper did not want to give up his clinical work. His appointment in Prague was delayed until 9 September, 1930, due to a highly controversial court case (see below). Arnold Pick (1851–1924) had held this position from 1886 until 1921, and Otto Pötzl (1877–1962) from 1922 until 1928, when he returned to Vienna [3, 5, 6]. Gamper was appointed to the State Board of Health in 1932. He declined the Chair in Königsberg in the same year, Chairs in Innsbruck and Graz in 1935 and was considered for the Chairs in Leipzig and Berlin in 1938 [6–8]. He served as Dean of the German Medical Faculty at the Charles University from 1935 to 1936, and became Head of the Student Medical Service, which he reorganized. Institutional improvements and personal advantages were promised in Prague, but then withheld and half-heartedly granted in June 1937 [6–8]. He was honoured with memberships of the German Society of Sciences in 1935 and the Leopoldina in 1936 [7, 8].

The conditions for patients, staff and students at the Department of Neuropsychiatry were unsatisfactory in every respect. Gamper worked to improve the situation and established a specialized ward for patients with neurological diseases in 1934. Asked on 19th April, 1938, the very day he went on holiday, not to forget the programmatic paper he had promised to write on “Chair on Neuropsychiatry—how it was, how it is and how it should be”, he allegedly assured the questioner that he was carrying it in his suitcase as he got into his car [9–11].

## Science

### Innsbruck I

Table 1 shows the range of Gamper’s scientific contributions. His first paper, prepared during his first year as an assistant at the Department of Neuropsychiatry and co-authored with K. Skutetzky, examined cerebrospinal fluid (CSF) changes in patients with syphilis. Several consecutive papers dealt with war-related lesions of the spine and brain. Gamper kept his interest in CSF and in trauma of the central nervous system until his death. In 1923, he published a study on postencephalitic rigidity, his first paper on movement disorders [5, 6].

**Table 1:** Gamper’s published scientific work covers a broad spectrum of topics combined with consistence and continuity in several areas

Topics year	CSF and serum	Spine and trauma	Movement	Alcohol	Inflammation	Neoplasm	Other
1913	Skutetzky						
1914							
1915		2 papers					
1916							
1917							
1918							
1919							
1920							Involutional Psychoses
1921							
1922	Chiari						
1923							
1924			Untersteiner				Arhinencephaly (presentation)
1925							
1926							Arhinencephaly (two major papers)
1927				Gruber			Trichinosis (with Gruber)
1928							Endemic Cretinism
1929		Stiefler				Stiefler	M. Recklinghausen (two papers)
1930					Stiefler		Halsmann case (expert opinion)
1931							Halsmann case; diencephalon
1932	Král	Král					
1933	Král		Kubik				“Profundol” sleeping pill (with H. Horn)
1934	Král						
1935	Král						Memorandum I. (for II. see text)
1936							
1937	Král	Stiefler					
1938							

The names of Gamper’s co-authors are mentioned (data from Gamper’s CV in [9, 10]; and from [5, 25]; Google Scholar; PubMed)

**Fig. 1:**
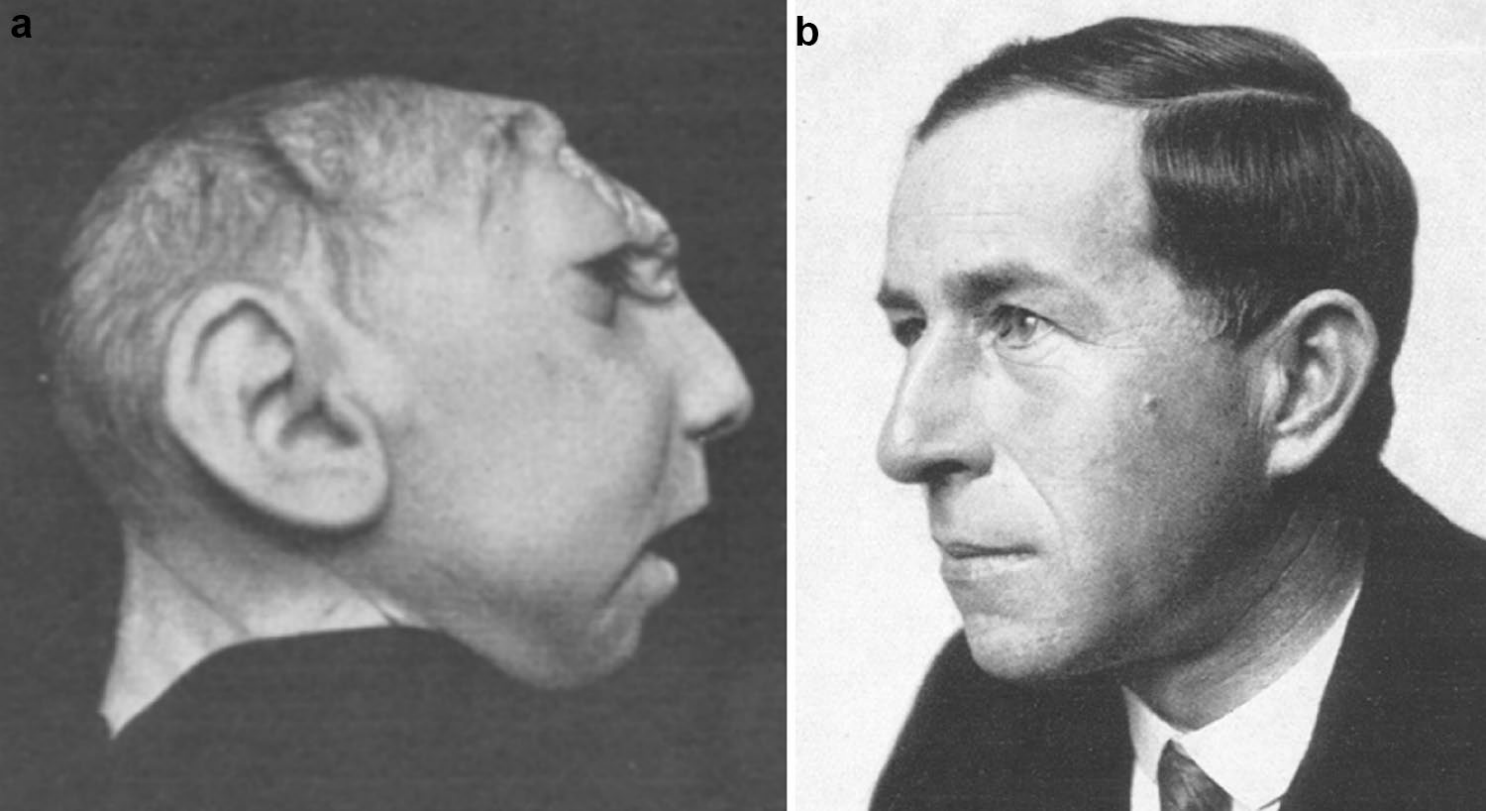
**a** and **b** the anencephalic child (Nannerl) and Eduard Gamper (from Gamper, 1926 [12]; from Stiefler, 1938 [29])

During the same year, he came to hear about a three month-old girl, with anencephaly, that came to be known as “Gamper’s midbrain-being”. The girl, Nannerl, blind and very weak, had been born on March 8, 1923, as the third illegitimate child of a 27-year-old maidservant [12]. Gamper convinced her mother and the obstetrician in charge of her case that he would take care of the child with all rights and duties. She was brought to him on June 7, 1923, a cold and rainy day. During the following week, Gamper and colleagues made diligent observations on her spontaneous behaviour and carried out a large number of tests, all documented in writing, with numerous photographs and in one of the very first medical films. In his detailed description, Gamper emphasises that the child was treated well and that great efforts were made to keep her warm. However, she developed fever on day 6 and died of pneumonia on day 7 after the transfer (Fig. 1) [12].

### Munich

Gamper used a large part of his time at the Kaiser Wilhelm Institute of Psychiatric Research with Walther Spielmeyer for the examination of Nannerl’s brain. The first publication in 1926 with 45 figures was 81 pages long and described the neuroanatomical findings in great detail. A publication on clinical findings with 38 figures and 71 pages followed in the same year. Gamper found that Nannerl’s spontaneous behaviour and reflexes were nearly normal for a child of her age: she slept and woke up, lolled, yawned, suckled, smiled and showed other typical reactions to acoustic, positional and haptic stimuli. On pressing the lower limbs in a supine position, she flexed her trunk (Gamper’s sign, indicative of severe cortical brain damage) [12–14].

### Innsbruck II

After his return, Gamper published two papers with Georg Gruber, one on trichinosis and a pivotal study on polioencephalitis haemorrhagica superior in chronic alcoholism, which laid the ground for his subsequent development of the neuropsychiatry of memory problems [15]. Most of the patients died within two weeks of disease onset. Gamper observed that the mamillary bodies were always affected, irrespective of the variable involvement of other brainstem areas. Therefore, he concluded that they must represent essential nodes for memory formation. A lesion of the Corpora mamillaria, which are normally connected with other midbrain, thalamic and neocortical areas, would therefore permit wakeful perception without leading to an increase of experience (“Erlebniszuwachs”) [15]. A number of invited textbook contributions and other influential papers followed.

On September 10, 1928, Morduch Max Halsmann (1880–1928), a 48-year-old, energetic and wealthy Jewish dentist from Riga, fell to his death during an exhausting hiking tour in the Alps he had taken with his son Philipp Halsmann (1906–1979). The circumstances were considered suspicious and additionally were touched by a climate of burgeoning antisemitism. Philipp, who had initially been acting rationally and responsibly, gave confusing evidence while under pressure during the criminal investigation in Innsbruck and at his trial, where he came across as arrogant and was judged to have been the murderer of his father. On October 16, 1928, he was sentenced to 10 years of hard labour. The case gained immense political weight with a wealth of opinions and authorities from all fields of science and society involved [12–14].

Gamper was chosen to represent the Innsbruck Medical Faculty at a re-trial in 1929. His testimony had to answer the question whether Philipp Halsmann could have forgotten the exact distance between himself and the victim, when his father had slipped down the slope. Gamper and colleagues opined that this was not possible at all and that the shock and painful experience of seeing his father fall, should have led to an even stronger memory formation. They mentioned repression (“Verdrängung”) as a possible obstacle in the way of proper recall, but such an idea from uninitiated personnel was gracelessly dismissed by Sigmund Freud (1856–1939) himself [19]. Gamper and colleagues further suggested that Philipp might have attacked his father in a rage caused by severe physical exhaustion. During the second trial, the jury followed this interpretation and felt that this was a case of manslaughter rather than murder so that the verdict was reduced to four years of hard labour [16–18].

Sigmund Freud, Albert Einstein (1879–1955), Thomas Mann (1875–1955) and many others voiced their strong views on “Austria’s Dreyfus Affair”. Philipp Halsmann was pardoned in October 1930 after he had contracted tuberculosis in jail [16, 18]. He was expelled from Austria, befriended the surrealist painter Salvador Dali' (1904–1989) in Paris. Halsmann soon became a world renowned art and fashion photographer for the magazines Vogue and Life, for which he produced 101 covers. Philipp Halsmann gained fame for his portraits, especially for those in which he made the portrayed jump (Marilyn Monroe, the Windsors, Richard Nixon, …). Halsmann also developed a philosophical discipline he frivolously called “jumpology” [20].

### Prague

Gamper’s arrival in Prague was delayed due to the Halsmann case and formal issues. There he found a number of experienced colleagues in or associated with the Department, some of them prolific scientists as Bruno Fischer (1888–1972) and Oskar Fischer (1876–1942), Franz T. Münzer (1895–1944), Otto Sittig (1886–1944), and Adalbert Král (1903–1988), his most prolific collaborator. Together with Hedwig Horn (born 1907), Gamper carried out an open label trial with a new animal-tested barbiturate-based sleeping pill (Profundol) on 66 patients with affective disorders, schizophrenia, senile brain diseases, neurosyphilis, postencephalitic and other conditions, all of them suffering from sleeping problems. The results were presented neatly and overall efficacy was seen as favourable.

Gamper was a gifted teacher popular with students and colleagues. He made great efforts to learn the Czech language within a short period of time. He allegedly sent several patients to Vienna for surgical interventions at his own expense. The particular situation in Prague, with one Czech und one German University side by side, caused numerous clinical, psychological, structural and economic problems for anyone involved. It was viewed quite critically by Aubrey Lewis, a prominent visitor in 1937 [21]. Gamper somehow managed to be on good terms with all parties and was hopeful until the end that conditions for the care and science in budding neurology and in neglected psychiatry could be improved [4, 7, 22, 23]. The last papers printed during Gamper’s life dealt with unnatural death due to cerebral concussion and choking [7, 23, 24]. His long second memorandum (Table 1) on the future of the Department of Neuropsychiatry in Prague was rescued from the lake Walchensee and published posthumously [11].

## The accident

### Reports

Breaking news from Wednesday 20 April, 1938, reported that a car had slipped over a steep cliff into the Walchensee next to the Post Hotel in the late afternoon. A cashier had observed the accident, immediately alarmed further staff and called in the nearby pioneers and members of a sports club. The automobile first floated on the surface, but immediate attempts to open the doors and break the windows to save the passengers were unsuccessful. The car sank six to eight metres to the bottom of the lake. Pioneers and numerous people worked hard for several hours until they were able to pull the car to the shore at 06:40 p.m.. The car with the license plate P-476 was a Tatra-75 6-seater with a front motor, 1700 cc and a top speed of 100 km/h. The victims were soon identified as “a doctor and his wife” in local [24] and international newspapers [26–28]. Their fox-terrier went unmentioned in all reports but one [28].

Weather forecasts on 20 April, 1938, had already warned of cold fronts, causing snowfalls at the end of April [24]. Dense snow-flurries were considered as the most likely explanation for the tragic accident [6, 24, 26–28].

Eduard Gamper, a Roman Catholic, and his wife Helene, a protestant, were both buried on 27 April, 1938, in Breitenwang near Reutte in Austria (with the generous permission from both churches). The large crowd attending the funeral included the German envoy to Prague, numerous colleagues and collaborators from Prague and learned societies, and also the local NSDAP-organisation from Reutte [24].

### Condolences and obituaries

More than 80 official telegrams and letters of condolence are conserved in the files of the Charles University Archives and Czech Academy of Science Archives [7, 8]. They came from the president of the Czechoslovakia, the German envoy to Czechoslovakia, the Cathedral Chapter, Czech and Prussian Academies of Science, Rockefeller Foundation, and many Czech, German and international universities.

One obituary by Helmut Scharfetter (1893–1979) published in the Archiv für Psychiatrie und Nervenkrankheiten [23] mentioned that there was “a second Gamper”, with cloudy, self-tormenting hours, when he appeared to doubt in himself, with no hope for future, tormented by presentiments of death. After an enthusiastic endorsement of Austria now being part of a larger Reich, another obituary by Georg Stiefler (1876–1939) from the Deutsche Zeitschrift für Nervenheilkunde [29] describes Gamper as a safe driver probably overwhelmed by particularly challenging snowfall. It also mentions a detail which helps to understand initial confusion regarding the Gampers’ destination: their car had hit a big log and made a 180 degree turn before it went over the cliff. Otokar Janota jr. (1898–1969)[4] praised Gamper’s conscientious attitude and diligent work as a scientist and clinician, who disliked vague and contrived theories. *“… He was much sought after as an excellent diagnostician and doctor. … He soon fell in love with Prague and also had the best contacts with Czech psychiatrists and neurologists. He was well informed about our work and watched with interest where and what was good. Recently, he has also dealt with the idea of gathering Czech and German neurologists and psychiatrists in joint scientific meetings. …*” The obituary by Král in the *Monatsschrift für Psychiatrie und Neurologie* provides most details of Gamper's scientific work and is otherwise rather inconspicuous [25].^1^

### Rumours and the evidence

Three suspicions have to be dealt with. First, it was assumed that Eduard Gamper had been dismissed from university due to Aryanization [31, 32]. Second, potential suicidal tendencies due to personality and life events were suggested [23]. Third, it was claimed that the Gampers’ car had been derailed and pushed off the road by a steel rope strung across a narrow Alpine road next the steep shores of the Walchensee [35, 36].

We have studied the literature including Adolf Hitler’s (1889–1945) detailed itinerary before the event [37], retrieved local and international newspapers [24, 26, 27] from the days after the event, visited and corresponded with archives, consulted international and local historians, the German weather service, identified and inspected to site of the accident. This is what we found:

In contrast with what has been suggested in one historical publication about Charles University [31], and quoted in a medical paper [32], Gamper was not at risk of losing his position because of racial and political issues. Academic and official representatives attended his funeral and he was a full member of the Medical Faculty at Charles University until his death on 20 April, 1938 [9, 10].

No evidence for a depressive episode can be found. Gamper had demonstrated his prowess, resilience and idealistic spirit for many years. He appeared in good spirits when he left Prague [6] and carried a nearly completed paper with him, which laid out the perspective for better treatment of patients. Gamper travelled with wife and their favourite dog. They were looking forward to seeing his elderly mother and extended family.

The multitude of voices from newspaper and radio messages do not appear orchestrated or manipulated. The reports describe the frantic efforts of many witnesses trying to rescue Eduard Gamper and his wife Helene [6, 24, 26–28].

A steel rope strung across the road at the Walchensee first appeared in writing many years after the accident [35]. This, and the Gestapo as likely culprits under the cover of an official version offering ice on the road, plus Gamper having been summoned to examine Hitler in Berchtesgaden before his car went over a cliff on his return, were duly mentioned years later [36]. Now Adalbert Král has been identified as the source of this narrative (A. Kertesz, pers. communication), which is otherwise unsubstantiated. The spot in plain sight and immediate vicinity of the Post Hotel would have been an unlikely choice for a trap or attack. Nothing had been noticed and reported by the large crowd involved in the sustained rescue efforts. According to the official weather data, a highly exceptional amount of 22 cm of fresh snow fell at the Walchensee between 20 and 21 April 1938—while Adolf Hitler celebrated his 49th birthday in Berlin watching the premiere of the Olympia-movie together with Leni Riefenstahl (1902–2003) [37].

## Epilogue

According to our current ethical standards some of Gamper’s studies may deserve critical reconsideration. There is however little doubt, that the adopted infant received the best possible care available at the time. Gamper’s studies on polioencephalitis haemorrhagica superior paved the ground for our understanding of limbic circuits as being essential for the formation of declarative memory. The expert opinion presented at the Halsmann trial rationally reflected the best clinico-pathologic understanding of memory function in the 1920s. Early systematic observation of the effects and side-effects of psychotropic drugs, also in patients with manifest neuropsychiatric disease, had been common academic practice in the first half of the last century, but rarely presented in such detail.

Gamper’s research betrays a good understanding of what can be resolved with the best methods available at his time. Gamper himself was one of the last versatile neuropsychiatrists, highly accomplished, but with an agenda largely unfinished.

Eduard Gamper shares the fate of drowning with neuropsychiatrist Bernhard von Gudden, who died in 1886 together with Ludwig II. in the Starnberg Lake, not far away from the Walchensee [38]. Gamper also shares the fate of a deadly traffic accident with Carl Wernicke, whose eponymous disease he studied, and who was run over by a timber cart during a cycling tour in 1905 [5].

## Data Availability

See references.

## Footnotes

1 Vojtech Adalbert Král survived the concentration camp of Terezin [30]. In 1947 he published a scientific paper about an endemic encephalitis between autumn 1943 and May 1944, which affected 978 prisoners whom he had treated. The disease showed an ophthalmoplegic-somnolent and a cerebellar variant, both with low mortality. The paper is written in a technical prose and an almost detached manner, except: “Unfortunately it will be impossible to follow up the majority of these cases as they have been transferred from Terezin to other places, where the greater part of them perished” [33]. Král later wrote seminal papers on issues around mild cognitive impairment [e.g. 34].
